# Naturally Acquired Resistance to *Ixodes scapularis* Elicits Partial Immunity against Other Tick Vectors in a Laboratory Host

**DOI:** 10.4269/ajtmh.20-0776

**Published:** 2020-11-30

**Authors:** Geoffrey E. Lynn, Husrev Diktas, Kathleen DePonte, Erol Fikrig

**Affiliations:** 1 Department of Internal Medicine, Section of Infectious Diseases, Yale University School of Medicine, New Haven, Connecticut;; 2 Howard Hughes Medical Institute, Chevy Chase, Maryland

## Abstract

In many regions where ticks negatively impact public health or economic production, multiple medically important tick species may have overlapping geographic distribution, and in North America, this includes members of *Ixodes*, *Dermacentor*, and *Amblyomma* genera. Acquired tick resistance is the process by which some animals develop an immune response against feeding ticks after one or more exposures. This form of immunity can restrict the ability of ticks to feed and may inhibit transmission of pathogens. Likewise, many proteins present in tick saliva are conserved among tick species, and prior studies have reported cross-protective host immunity against certain combinations of ticks. In this study, we used a guinea pig model to assess whether host resistance against *Ixodes scapularis* could confer protection against two other medically important tick vectors, *Dermacentor variabilis* and *Amblyomma americanum*. Tick challenges using nymphs were used to induce host resistance against a primary species, followed by additional challenge using a secondary tick species. Tick attachment to hosts and engorgement weights were reduced significantly for *D. variabilis* and *A. americanum* feeding on *I. scapularis*–sensitized hosts. Reciprocally, *I. scapularis* engorgement weights were reduced to a lesser extent, and attachment was unaffected when feeding on hosts sensitized with either *D. variabilis* or *A. americanum*. These results indicate that immunity against *I. scapularis* could potentially be exploited for use in an anti-tick vaccine targeting multiple tick species and their associated pathogens.

## INTRODUCTION

Ixodid ticks are ectoparasites capable of transmitting a broad variety of human and veterinary pathogens. The black-legged tick, *Ixodes scapularis*, is a primary vector for at least seven pathogens affecting humans, including the agent of Lyme disease, the most common vector-borne illness in the United States and Canada.^1^ One or more human pathogens can be transmitted during each of the larval, nymphal, or adult stages, although most of the *I. scapularis*–borne infections occur in close proximity to peak periods of nymphal activity.^2–4^ Although the geographic distribution of tick vectors and the diseases associated with them continue to increase,^5^ effective vaccines to prevent most tick-borne diseases are lacking.

Acquired tick resistance (ATR) occurs when repeated tick infestations result in a host immune response that impairs tick feeding or causes outright tick rejection.^6^ This phenomenon has been demonstrated for cattle as well as in several laboratory animal species and is especially robust for guinea pigs.^7–9^ Although the mechanisms are not fully understood, ATR involves both humoral and cellular immune components. Demonstrated or hypothesized contributors include keratinocytes, natural killer cells, dendritic cells, T cells, B cells, neutrophils, mast cells, basophils, eosinophils, endothelial cells, cytokines, chemokines, and complement and extracellular matrix.^10–13^ Acquired tick resistance may impact ticks in a variety of ways, including premature detachment from or prolonged attachment to hosts, reduced engorgement weight, reduced molting success, and reduction in fecundity. Interestingly, laboratory strains of mice do not readily develop robust ATR against some tick species, including *I. scapularis*, which contributes to the challenges of studying this phenomenon. Since its discovery, ATR continues to hold strong interest within the field of public health because this type of immunity has been shown to inhibit tick-to-host transmission of multiple tick-borne pathogens, including *Borrelia burgdorferi*.^9,14–20^ In addition, there is strong suggestive evidence that cutaneous hypersensitivity to *I. scapularis* in humans is associated with reduced risk of Lyme disease.^21^

Although they effectuate comparatively less public health impact than *I. scapularis*, American dog ticks (*Dermacentor variabilis*) and lone star ticks (*Amblyomma americanum*) are either important vectors or suspected vectors for a number of tick-borne diseases in North America, which include rickettsioses, ehrlichioses, Q fever, tularemia, and the newly recognized Heartland and Bourbon viruses.^22–29^ In the context of ATR, cross-species resistance and cross-protective immunity are descriptors for when sensitization of a host animal against a primary tick species elicits a protective immune response against subsequent feeding by a different species of tick. Multiple studies have reported cross-protection; however, there appears to be considerable heterogeneity in interspecies interactions, and cross-protective ATR attained using *I. scapularis* as the primary route of sensitization has not been documented.^8,11,30^ Our purpose was to evaluate whether naturally acquired immunity against *I. scapularis* nymphs could confer protection against two other medically important tick species, and if so, to determine whether the effect was multidirectional.

## MATERIALS AND METHODS

### Ethics statement.

All research involving animals was performed in accordance with the recommendations of the Guide for the Care and Use of Laboratory Animals of the NIH and approved by the Yale Institutional Animal Care Committee (YIACUC) under protocol 2018-078941. Experiments using animals were conducted in a biosafety level 2 facility according to YIACUC rules.

### Ticks and guinea pigs.

Because *I. scapularis* nymphs are the most significant concern for public health, this life stage was chosen for experiments. Along with *A. americanum* and *D. variabilis*, *I. scapularis* ticks used in this study were sourced from specific pathogen-free colonies maintained at Oklahoma State University (Stillwater, OK) and housed at 23°C at 90% humidity under a 14-hour light, 10-hour dark photoperiod. Four- to 6-week-old female Hartley guinea pigs were purchased from Charles River (MA). The dorsa of guinea pigs were shaved before tick infestations to allow observation of tick attachment. To allow hosts to groom themselves, which in tick-immune guinea pigs is often a natural response to dermal inflammation occurring at the feeding lesions, ticks were allowed to attach freely without the use of feeding capsules. Partial shaving and/or temporarily replacing cut hair over *A. americanum* nymphs was a necessary adjustment to encourage attachment; however, within the first 3 hours of placement, some ticks moved to unshaved areas where they were not visible and could not be counted. Guinea pigs were anesthetized using a mixture of ketamine and xylazine, and between 26 and 28 nymphs were placed on the shaved dorsum of each individual infested with *I. scapularis* or *D. variabilis*. Because some *A. americanum* ticks preferred to attach outside of shaved areas, 37 nymphs were applied to individual guinea pigs infested with *A. americanum* to ensure an adequate number of ticks attached in the shaved regions where duration of attachment could be easily monitored. At least 14 nymphs were recorded as attached in the shaved region of each guinea pig at 2 hours following infestation. Only ticks attached within the shaved region were included in attachment calculations.

Ticks were allowed 3–4 hours to attach, after which tick attachment numbers were recorded. Guinea pigs were housed in sealed, wire-bottom cages as described previously in the study by Narasimhan et al.,^31^ and daily monitoring of guinea pigs and ticks was performed to record tick attachment, collect unattached ticks, and observe any erythema. Cages were cleaned each day, and pan water was changed, ensuring any detached ticks were removed.

**Figure 1.**
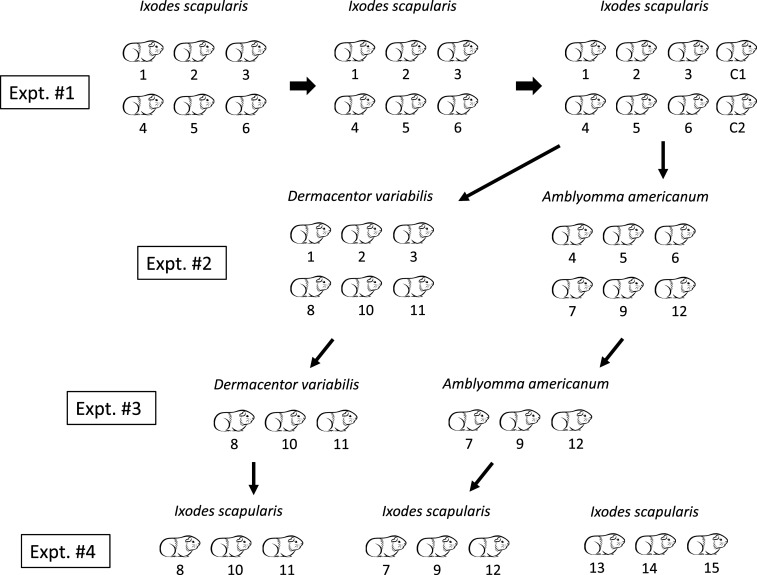
Summary of guinea pig groups used in tick challenge experiments.

Beginning at 72 hours after infestation, detached ticks were collected from pans at 24-hour intervals, where they were immediately submerged in H_2_O and surface-cleaned with a soft paint brush, followed by drying on filter paper before weighing. After ticks were counted and individual weights were recorded, engorged ticks were incubated at 23°C in clean, vented polystyrene tubes under 90% humidity and a 14-hour light, 10-hour dark photocycle. After a period of 10 weeks, successful conversion to the adult stage was assessed. Guinea pigs exposed ticks on multiple occasions (Figure 1) and were allowed a resting interval between 2 and 4 weeks between tick challenge experiments, beginning once all ticks had detached, and each replicate included in a treatment group received the same rest period. Previous experiments and personal experience have demonstrated that ATR in guinea pigs persists well beyond 12 weeks.^7,32^

For tick tissue collection, guinea pigs were infested with nymphs as described earlier, and partially engorged ticks were later removed from guinea pigs under isoflurane anesthesia using a fine-tipped forceps. Following manual removal, ticks were kept on ice until they could be dissected in cold phosphate-buffered saline (PBS) using a sterile needle. Salivary glands were carefully removed, washed in cold PBS, and frozen at −80°C until later use. Extracts were then further disrupted by two 30-second cycles of ultrasonication followed by cold centrifugation for 10 minutes at 10,000 g to separate cellular debris. Extracts collected at 24 hours, 48 hours, and 72 hours post-infestation were pooled for ELISA assay.

### ELISA assessment of salivary gland extract-specific IgG levels.

Ninety six-well plates were coated with 250 ng of salivary gland extract. Guinea pig sera collected 2 weeks after tick challenge for following tick challenge experiments 1 and 3. Each serum was diluted at 1:500 and 1:5,000 before use for primary labeling. horseradish peroxidase (HRP)-conjugated goat anti-guinea pig IgG with 3,3′, 5,5″-tetramethylbenzidine (TMB) substrate solution (ThermoFisher Scientific, Rockford, IL) was used as secondary labels. ELISA procedures were performed as previously described.^31^ In the absence of a commercially available label for guinea pigs, IgE levels were not measured.

### Statistical and graphical analyses.

Tick recovery percentage was calculated as total ticks collected per guinea pig divided by total ticks placed on guinea pig, multiplied by 100; molting success was calculated by dividing molted ticks by ticks collected, multiplied by 100. For tick engorgement weights, percent replete ticks recovered, and percent molting success, significance of difference was calculated using the Mann–Whitney test for comparisons between two groups. One-way analysis of variance with Tukey’s multiple comparison test was used for comparisons between three groups, and *P* < 0.05 was considered statistically significant. Statistical significance tests were performed using Prism 8 (Graphpad Software, San Diego, CA). Prism and Adobe Photoshop (Adobe Inc., San Jose, CA) were used to generate figures.

## RESULTS

### Primary resistance against *I. scapularis* (experiments 1 and 2).

#### Tick challenge experiment 1.

**Figure 2.**
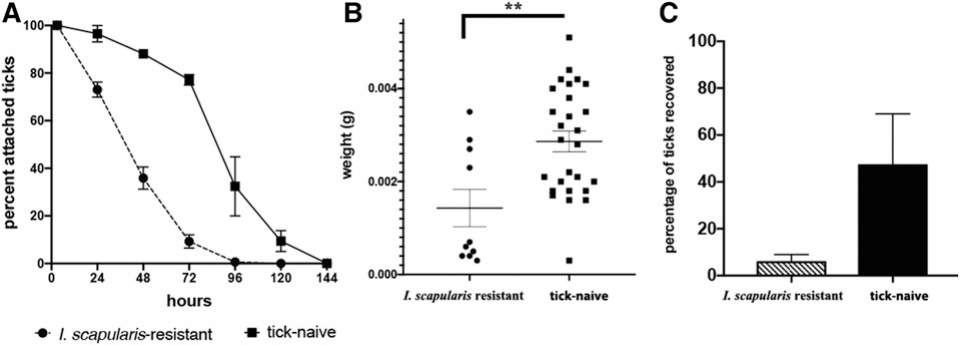
Guinea pigs developed acquired tick resistance against *I. scapularis* nymphs. (**A**) Rate of tick detachment from *I. scapularis*–resistant or tick-niïve guinea pigs; (**B**) engorgement weights of individual nymphs; (**C**) percent recovery of nymphs. Error bars represent means ± SEM. Significance of differences assessed in (**B**) and (**C**) by the Mann–Whitney test (**P < 0.01). *I. scapularis* = *Ixodes scapularis*.

Acquired tick resistance was demonstrated for six guinea pigs challenged with *I. scapularis* nymphs for a third time. Tick attachment percentage and mean engorgement weights were strongly reduced (2.9 µg versus 1.4 µg), and fewer ticks were recovered on average from the six tick-resistant guinea pigs (5.5%) than from the two tick-naive guinea pigs (47.5%), although the difference was not statistically significant, *P* = 0.071 (Figure 2A–C). Pronounced cutaneous erythema at the bite sites was observed on all guinea pigs during secondary and tertiary tick challenges.

#### Tick challenge experiment 2.

**Figure 3.**
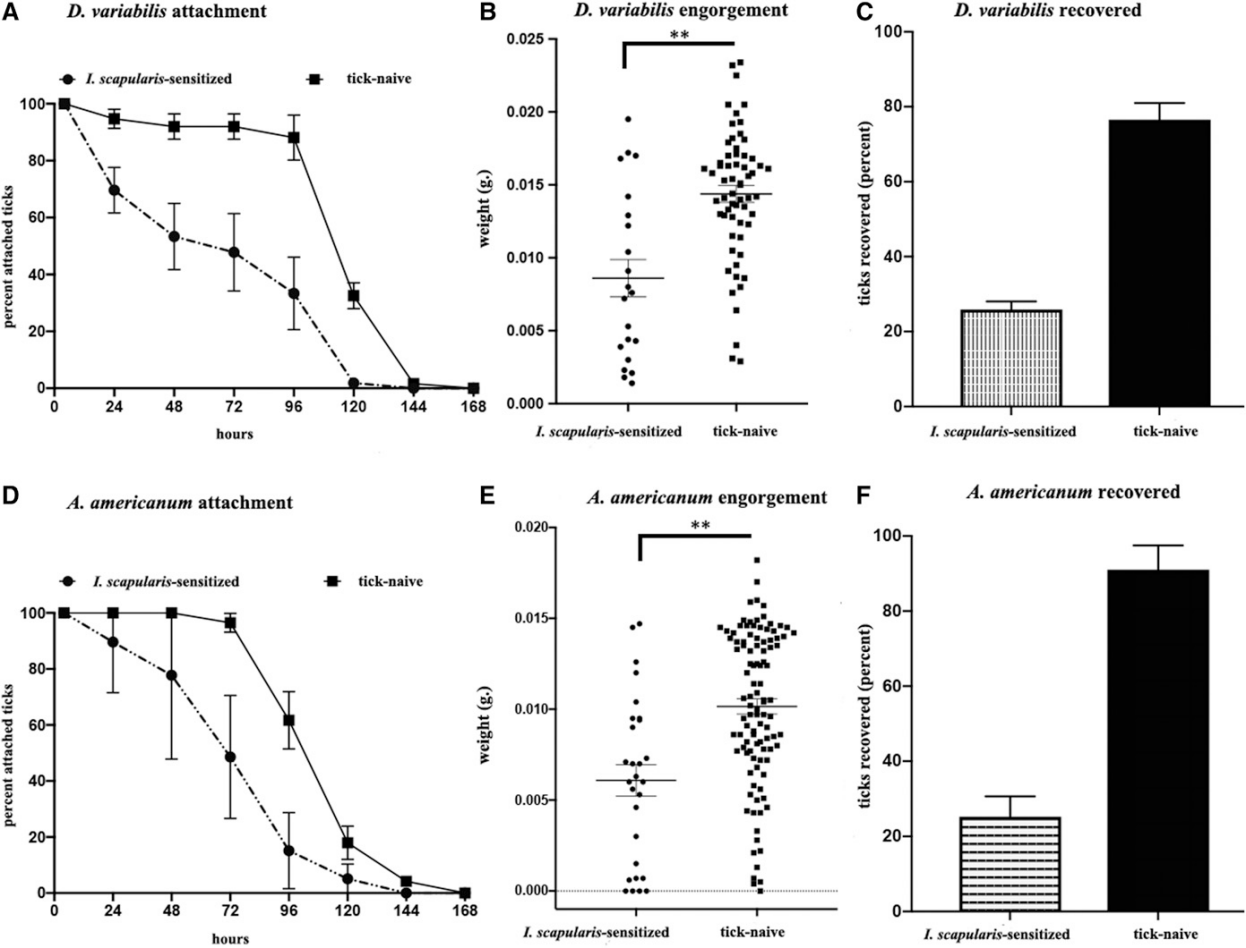
Cross-protective effects on *D. variabilis* and *A. americanum* nymphs feeding on hosts with acquired tick resistance against *Ixodes scapularis* nymphs. (**A**) Rate of tick detachment by *D. variabilis* nymphs; (**B**) engorgement weights of individual *D. variabilis* nymphs; (**C**) percent recovery of *D. variabilis* nymphs; (**D**) rate of tick detachment by *A. americanum* nymphs; (**E**) engorgement weights of individual *A. americanum* nymphs; (**F**) percent recovery of *A. americanum* nymphs. Error bars represent means ± SEM. Significance of differences assessed in (**B**), (**C**), (**E**), and (**F**) by the Mann–Whitney test (**P < 0.005). *A. americanum* = *Amblyomma americanum*; *D. variabilis* = *Dermacentor variabilis*.

Attachment percentage of *D. variabilis* nymphs feeding on *I. scapularis*–resistant hosts was reduced relative to naive hosts over the span of tick feeding (Figure 3A), and the effect was most apparent during the first 48 hours following infestation. Engorgement weights were reduced by 40.2% relative to controls (8.6 µg versus 14.4 µg; Figure 3B). We also observed that the percentage of ticks recovered was lower for ticks placed on resistant hosts (25.9% compared with 74.1%) (Figure 3C). There was no clear, consistent difference in erythema between naive hosts and tick-resistant hosts. Of the ticks collected, 66.7% of nymphs from resistant hosts successfully molted into adults, whereas 87.1% from tick-naive hosts successfully molted, a difference that was not statistically significant.

*Amblyomma americanum* nymphs feeding on *I. scapularis*–resistant hosts showed a greater rate of premature detachment within the first 72 hours after infestation (Figure 3D) and 40.1% reduction in engorgement weight (6.1 µg versus 10.2 µg) relative to nymphs collected from tick-naive hosts (Figure 3E). Tick recovery was low (24.3%) for resistant hosts compared with tick-naive hosts (94.6%) (Figure 3F). Similar to our observations for *D. variabilis*, there was no clear, consistent difference in redness among tick-resistant and tick-naive hosts. Of the nymphs collected from tick-sensitized hosts, 53.6% molted into adults, whereas 85.7% of nymphs from tick-naive hosts successfully molted. This difference was not statistically significant.

### Primary resistance against *D. variabilis* or *A. americanum* (experiments 3 and 4).

#### Tick challenge experiment 3.

**Figure 4.**
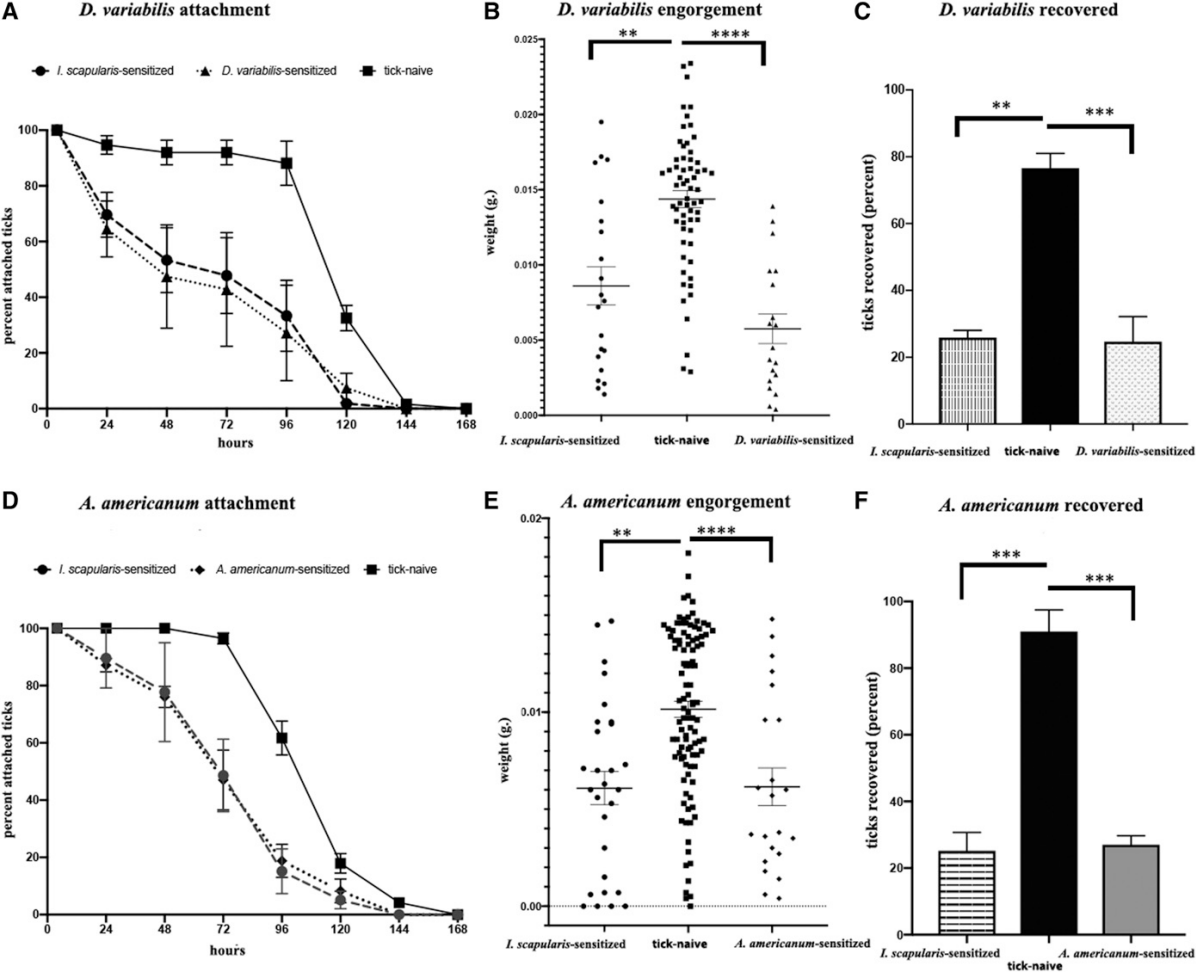
Host resistance to heterologous tick challenge compared with homologous tick challenge. Results for ticks fed on *Ixodes scapularis*–sensitized hosts are depicted in red, green for *D. variabilis*–sensitized hosts, and blue for *A. americanum*–sensitized hosts. (**A**) Rate of tick detachment by *D. variabilis* nymphs; (**B**) engorgement weights of individual *D. variabilis* nymphs; (**C**) percent recovery of *D. variabilis* nymphs; (**D**) rate of tick detachment by *A. americanum* nymphs; (**E**) engorgement weights of individual *A. americanum* nymphs; (**F**) percent recovery of *A. americanum* nymphs. Error bars represent means ± SEM. Significance of differences assessed in (**B**), (**C**), (**E**), and (**F**) by one-way ANOVA with Tukey’s multiple comparison test (*P < 0.05, **P < 0.005, ***P < 0.001, ****P < 0.0001). *A. americanum* = *Amblyomma americanum*; *D. variabilis* = *Dermacentor variabilis*; *I. scapularis* = *Ixodes scapularis*.

Guinea pigs used as tick-naive controls in the previous experiment were challenged a second time with nymphs of the same tick species as during the primary sensitization. The effect of *D. variabilis* sensitization on *D. variabilis* attachment was similar to that observed on *I*. *scapularis*–resistant hosts, with a notable decline during the initial 48-hour period (Figure 4A). Mean engorgement weight (5.3 µg versus 14.4 µg, −63.1%) and percent recovered (24.7% versus 76.6%) were reduced compared with the primary challenge (Figures 4B and C), and all three animals showed strong redness at the site of tick attachment from 24 hours until ticks were no longer attached. Detachment from *D. variabilis*–sensitized hosts was only slightly better than that for *I. scapularis*–sensitized hosts, and differences were within the margins of error. Engorgement weights and percent recovered were lower for ticks from *D. variabilis*–sensitized hosts than those from *I. scapularis*–sensitized hosts, although this difference was not statistically significant.

Guinea pigs challenged a second time with *A. americanum* also demonstrated ATR, as expected. The tick attachment response was similar to *A. americanum* feeding on *I. scapularis–*sensitized hosts, including a clear effect within the first 72 hours (Figure 4D). Mean engorgement weight was reduced by 57.2% (4.4 µg versus 10.2 µg) compared with weight from primary sensitization of these same animals (Figure 3E); 27.0% of ticks from *A. americanum*–sensitized hosts were collected, compared with 91.0% from tick-naive hosts (Figure 4F). Guinea pigs were not shaved fully to skin level to encourage ideal tick positioning, and skin redness was not assessed. Tick detachment percentages for *A*. *americanum* nymphs feeding on either *I. scapularis*–sensitized or *A. americanum*–sensitized hosts were highly similar, and differences between these two groups were not statistically significant.

#### Tick challenge experiment 4.

**Figure 5.**
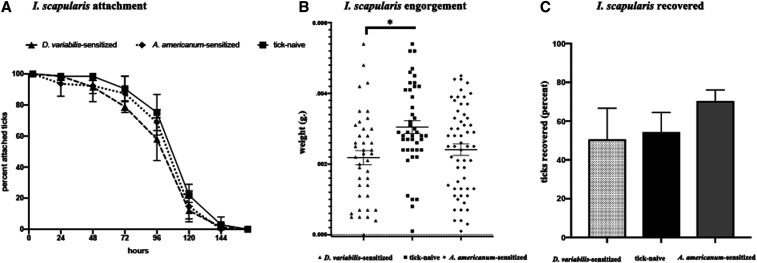
Cross-protective effects on *Ixodes scapularis* nymphs feeding on hosts with acquired tick resistance against either *Dermacentor variabilis* or *Amblyomma americanum* nymphs. (**A**) Rate of tick detachment by *I. scapularis* nymphs; (**B**) engorgement weights of individual *I. scapularis* nymphs; (**C**) percent recovery of *I. scapularis* nymphs. Error bars represent means ± SEM. Significance of differences assessed in (**B**) and (**C**) by one-way ANOVA with Tukey’s multiple comparison test (*P < 0.05). *I. scapularis* = *Ixodes scapularis*.

Attachment percentage of *I. scapularis* nymphs fed on hosts resistant to *D. variabilis* declined minimally relative to controls, whereas no difference was observed for ticks feeding on *A. americanum*–sensitized hosts (Figure 5A). Mean engorgement weights were 28.5% and 21% lower for ticks fed on *D. variabilis*–sensitized hosts (2.21 µg) and *A. americanum*–sensitized hosts (2.36 µg), respectively, relative to naive hosts (2.94 µg) (Figure 5B). Tick recovery percentage did not differ significantly among any of the treatment groups (50.6%, 70.4%, and 70.4%, respectively, from *D. variabilis–*, tick-naive–, and *A. americanum*–sensitized hosts) (Figure 5C). No clearly distinguishable differences in skin erythema were observed between resistant and control host groups. Molting success was low (between 30% and 40% for ticks from each of the three groups’ hosts), and these differences were not statistically significant.

### Specificity of seroreactivity to salivary gland extracts.

**Figure 6.**
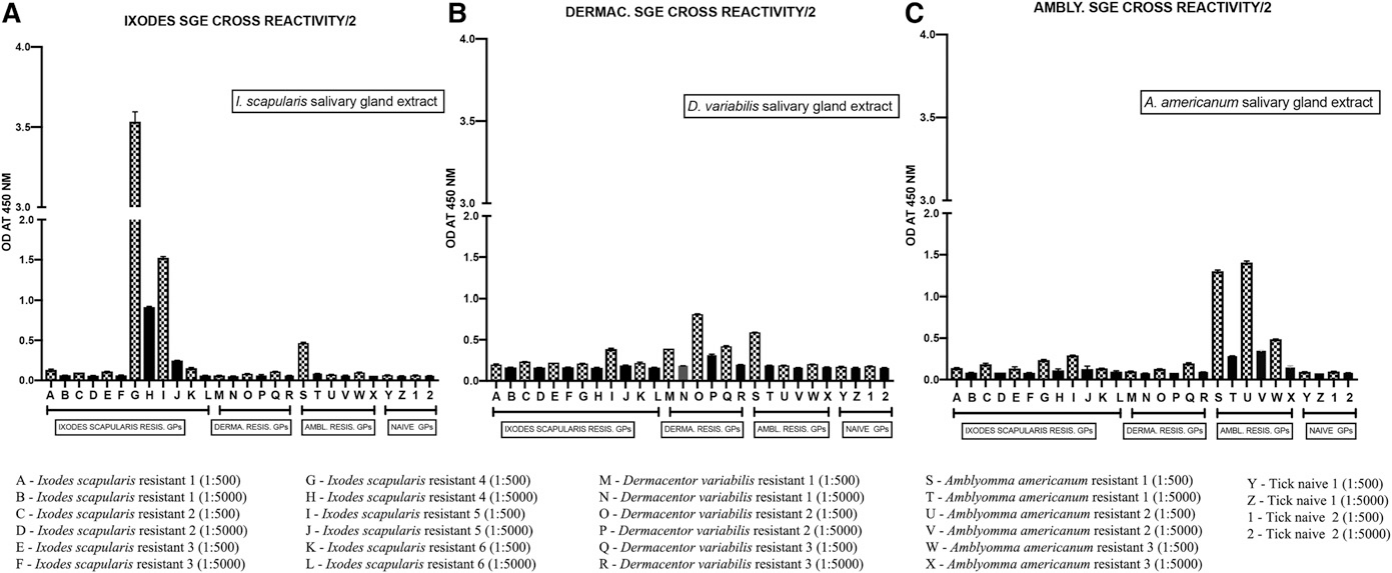
Serological response to salivary gland extracts measured by ELISA. Antisera from guinea pigs with at least two conspecific tick exposures, using tick challenges by one of the following species: *I. scapularis* (*n* = 6 guinea pigs, three tick exposures), *D. variabilis* (*n* = 3, 2 exposures), *A. americanum* (*n* = 3, 2 exposures), or tick naive (*n* = 2, 0 exposures), were assessed by ELISA for homologous reactivity and cross-reactivity to salivary extracts from (**A**) *I. scapularis*; (**B**) *D. variabilis*; or (**C**) *A. americanum.* Each serum was tested at two dilutions (1:500 and 1:5,000) positioned consecutively in the figure. *A. americanum* = *Amblyomma americanum*; *D. variabilis* = *Dermacentor variabilis*; *I. scapularis* = *Ixodes scapularis*.

Reactivity of anti-tick sera to SGE was highly variable, even among sera with the same series of tick exposures. In ELISA, antisera from only two of six guinea pigs with ATR against *I. scapularis* showed strong reactivity to SGE from this species (Figure 6A). Sera from each of three *D. variabilis*–sensitized guinea pigs did not react with *I. scapularis* SGE, whereas one of three *A. americanum*–sensitized sera reacted modestly. All three *D. variabilis* antisera showed modest reactivity against *D. variabilis* SGE (Figure 6B), whereas one of three *A. americanum* was slightly reactive, and *I. scapularis* antisera were minimally reactive with *D. variabilis* SGE. Two of three *A. americanum* antisera reacted strongly to conspecific SGE, and one modestly with *A. americanum* SGE (Figure 6C). Both *I. scapularis* antisera and *D. variabilis* antisera reacted minimally with *A. americanum* SGE.

## DISCUSSION

*Ixodes scapularis*, *D. variabilis*, and *A. americanum* are important vectors of disease for humans, companion animals, and livestock in North America and belong to tick genera that are distributed globally. These three species frequently coexist in nature and each stimulates ATR in guinea pigs, presenting an ideal model to study cross-species immunological interactions. In addition, guinea pigs like humans are incidental hosts for these tick species, and their immune responses, especially in the skin, are more approximate to those in humans than mice.^9,21,33^ In this set of experiments, we demonstrated that primary sensitization using a prostriate species (*I. scapularis*) conferred a partially protective effect against two metastriate ticks (*D. variabilis* and *A. americanum*) that were statistically significant. By each measure of feeding success quantified (tick attachment, engorgement weights, and recovery of replete ticks), metastriate ticks were negatively impacted by *I. scapularis*–specific ATR. Interestingly, data combined from separate experiments indicate that primary sensitization using *I. scapularis* conferred a comparable level of host protection against feeding *D. variabilis* and *A. americanum* as primary sensitization using the same species of metastriate tick did. Because these results were derived from two separate experiments, and *I. scapularis*–resistant hosts had already received two tick challenges, it is an imperfect comparison. However, previous studies reported small declines (approximately 8% and 13%) in engorgement weights for *A. americanum* larvae and adults feeding on thrice-challenged hosts compared with twice-challenged hosts,^8,22^ which suggests that an additional sensitization may not have altered the results of our comparison much. In addition, using *A. americanum* larvae, Brown and Askenase^11^ reported a 38.9% decline in engorgement weight from the first to the second challenge that was very similar to our results obtained using nymphs, and also suggest that our cross-species findings are likely to be applicable to larval stages. Conversely, we observed a lesser protective effect against feeding *I. scapularis* when hosts were sensitized first with either of the two metastriate species included in this study. Attachment and recovery of *I. scapularis* were largely unaffected by prior host sensitization with metastriate species, whereas engorgement weights were reduced, although much less so than prostriate sensitization impacted metastriate engorgement. These modest results are more similar to the previously reported 16% reduction in engorgement weight for larval *I. scapularis* feeding on guinea pigs sensitized with larvae of *Dermacentor andersoni*, a close relative of *D. variabilis*.^30^ The differences described here suggest that fundamental differences in the biology of feeding for the two lineages of hard ticks (prostriate and metastriate) may influence how host immunity against ticks develops.

One possibility is that saliva of either of the metastriate ticks is less immunogenic for guinea pigs than *I. scapularis*. Specifically, this may apply to the composition of attachment cement, which is a complex proteinaceous mixture secreted early in tick saliva with multiple functions, including adherence of tick mouthparts to host skin.^34–37^ Although it is relatively understudied, cement may also act as a depot in the skin for antigen presentation^38^ and has gained renewed research attention as of late.^39,40^ In 1966, Moorhouse demonstrated that the initial layer of cement (internum or core) shielding the hypostome, in addition to protein, contains lipids, and is followed by a secondary layer (cortex) that hardens around the core layer and is directly in contact with the host skin. The cortex comprises protein and carbohydrates. Whereas metastriate species produce both layers, it has been reported that certain prostriate ticks, including *Ixodes ricinus*, a sister species of *I. scapularis*, secrete only a core layer.^35,36,41^ This distinction could influence cross-protective immunity if a prostriate species were to constitutively present a lipoprotein cement layer to host immune defenses throughout the feeding period. However, in a metastriate tick, a more brief period of core layer exposure followed by concealment within a subsequent glycoprotein layer of cement could potentially limit both the window of cross-protective effect to the phases of feeding, as well as the intensity of host response induced by briefly exposed antigens. In support of this hypothesis, it has been reported that the major hemolipoprotein found in the saliva of a *Dermacentor* species binds to carbohydrates.^42^ However, there are a number of highly conserved proteins within the saliva of these three species^43^ that may have value as vaccine candidates.

A second potential factor that may influence the relative susceptibilities of tick species to ATR that has been discussed previously (McTier et al.^30^) is species-specific differences in the depth of penetration of tick mouthparts into host skin. This hypothesis corresponds with our observation that the species with the shortest mouthparts (*D. variabilis*) was the most susceptible to cross-protective ATR, whereas the species with the longest mouthparts (*I. scapularis*) was least impacted.^44,45^ Given that the quantity of cement and the structure of cement cones produced by specific tick species are thought to be related to the depth to which its mouthparts penetrate the host skin, these attributes are likely to play an integrated role in the development of ATR.

It is significant that in our tick challenges, the strongest observable effect on feeding as suggested by premature detachment occurred during the early phases of feeding following infestation. The biochemical composition of ixodid saliva has been shown to be highly dynamic throughout the multiday feeding process, where hundreds of proteins are differentially expressed at various time points.^14,43,46–48^ *Ixodes scapularis* proteins expressed during the first 24 hours of feeding have been shown to stimulate strong inflammatory reactions, resulting in tick rejection and inhibition of tick transmission of *B*. *burgdorferi*.^14^ In consideration with these findings, our data suggest that some tick antigens secreted early in the feeding process may include immunogenic, highly conserved antigens with the potential to disrupt pathogen transmission. Importantly, considerable variation exists among potential pathogens in the period between tick attachment and salivary transmission. The extent of this window is likely to influence how effectively a vaccine can disrupt transmission, as certain pathogens including *Borrelia miyamotoi* and Powassan virus may be transmitted to the vertebrate host within hours after tick feeding is initiated.^49^ For these and other rapidly transmitted disease agents, a robust immediate host immune response is likely to be necessary to impede transmission, whereas other tick-borne pathogens including *B*. *burgdorferi* and *Babesia microti* are transmitted inefficiently, if at all during the first 1 or 2 days following tick attachment, and may consequently be more susceptible to a slower onset of host immunity. Nevertheless, ATR may yet provide some benefit against certain rapidly transmitted pathogens, as was previously shown for *Francisella tularensis*.^16^

Although previous studies have described a humoral immune contribution to ATR, in this study, our ELISA did not provide evidence that this mechanism plays a role in naturally acquired cross-protection in guinea pigs. Somewhat surprisingly, only two of six sera from guinea pigs, all with demonstrated ATR against *I. scapularis*, reacted with conspecific SGE, and seroreactivity against conspecific SGE was also modest for the metastriate antisera included our study. One likely reason for this is that ticks are able to modulate host immune responses.^6,50,51^ A recent study by Xu et al.^52^ showed that a tick serine protease inhibitor suppressed adaptive immune components, including IgG2, and it is conceivable that in guinea pigs, low levels of humoral immunity, in concert with cellular immunity, may be sufficient to induce a cutaneous hypersensitivity response. Passive transfer experiments comparing tick rejection induced by either immune serum or peritoneal exudate cells/lymph node cells have shown that cellular immunity has a greater effect than humoral immunity, although the differences are inconsistent among tick species and, to our knowledge, have not been reported for *I. scapularis*.^11,32,53^ Furthermore, we have also previously shown that IgG response to tick saliva guinea pigs exposed to *I. scapularis* nymphs was minimal compared with that of mice, which developed much higher titers than guinea pigs despite their lack of resistance against tick feeding.^33^ Although it has been shown that IgE plays an essential role in immunity of mice, efforts to discern significant amounts of IgE in guinea pigs have been unsuccessful on account of currently limited capabilities of immunological reagents for this animal model.^31^ In addition, the extent that host grooming behavior associated with dermal inflammation contributes to ATR is not currently well understood, although a studying comparing feeding performance of free ranging and chambered ticks on resistant hosts would provide further insight.

In conclusion, the results of this study demonstrate that host immunity to *I. scapularis*, a prostriate tick, can confer a protective effect against metastriate ticks. This is important in North America, where nymphal *I. scapularis* are the primary vectors of tick-borne pathogens of humans and, as such, are a priority for vaccine development. Two additional medically important tick species, *D. variabilis* and *A. americanum*, can be found in many parts of the United States where they overlap with the geographical range of *I. scapularis*, and the endemic range of *A. americanum* is expanding north where it is in closer proximity with the other two species.^5,54^ Previous studies have shown that tick antigens can be used effectively to immunize hosts against multiple tick species.^55,56^ Our data provide evidence suggesting that a vaccine targeting *I. scapularis* antigens could be selectively developed to stimulate host resistance to, and potentially interrupt transmission of, pathogens by multiple tick species of medical importance. Further work on cross-protective ATR should address the cellular mechanisms of immunity in greater depth and evaluate specific antigens for broad applicability as immunogens.
